# Research progress on the antitumor effects of harmine

**DOI:** 10.3389/fonc.2024.1382142

**Published:** 2024-03-25

**Authors:** Yonghua Hu, Xiaoli Yu, Lei Yang, Gaimei Xue, Qinglin Wei, Zhijian Han, Hao Chen

**Affiliations:** ^1^ Key Laboratory of the Digestive System Tumors of Gansu Province, Department of Tumor Center, The Second Hospital & Clinical Medical School, Lanzhou University, Lanzhou, China; ^2^ The First Clinical Medical College, Gansu University of Chinese Medicine, Lanzhou, China; ^3^ Affiliated Hospital of Gansu University of Chinese Medicine, Lanzhou, China

**Keywords:** harmine, *Peganum harmala*, β-carboline alkaloid, antitumor effect, mechanism of action, derivatives

## Abstract

Harmine is a naturally occurring β-carboline alkaloid originally isolated from *Peganum harmala*. As a major active component, harmine exhibits a broad spectrum of pharmacological properties, particularly remarkable antitumor effects. Recent mechanistic studies have shown that harmine can inhibit cancer cell proliferation and metastasis through epithelial-to-mesenchymal transition, cell cycle regulation, angiogenesis, and the induction of tumor cell apoptosis. Furthermore, harmine reduces drug resistance when used in combination with chemotherapeutic drugs. Despite its remarkable antitumor activity, the application of harmine is limited by its poor solubility and toxic side effects, particularly neurotoxicity. Novel harmine derivatives have demonstrated strong clinical application prospects, but further validation based on drug activity, acute toxicity, and other aspects is necessary. Here, we present a review of recent research on the action mechanism of harmine in cancer treatment and the development of its derivatives, providing new insights into its potential clinical applications and strategies for mitigating its toxicity while enhancing its efficacy.

## 1 Introduction

As a multi-targeted and multi-mechanistic adjuvant therapy, traditional Chinese medicine offers the advantages of relatively few toxic side effects, mild efficacy, and the ability to sustain consistent medication (1, 2). As of 2019, approximately 25% of all newly approved anticancer drugs were naturally derived. Natural alkaloids with significant medicinal value are expected to play key roles in cancer treatment in the foreseeable future (3).

*Peganum harmala L.* has a wide range of pharmacological effects, including cardiovascular, neurological, gastrointestinal, antimicrobial, antidiabetic, osteogenic, immunomodulatory, menstruation-promoting, and antitumor activities, along with other pharmacological effects (4). More than 160 alkaloids, including β-carbolines, quinoline, quinazolines, and indoles, have been isolated from *P. harmala L* (5). β-carboline alkaloids are the most important constituents of *P. harmala L*. and are responsible for most of its pharmacological effects. Among these, harmine, harmaline, harmane, harmol, and harmalol are the most abundant and biologically active components. Harmine, the primary active alkaloid, accounts for 3.2% of the chemical constituents present in *P. harmala L*. roots (6).

**Figure 1:**
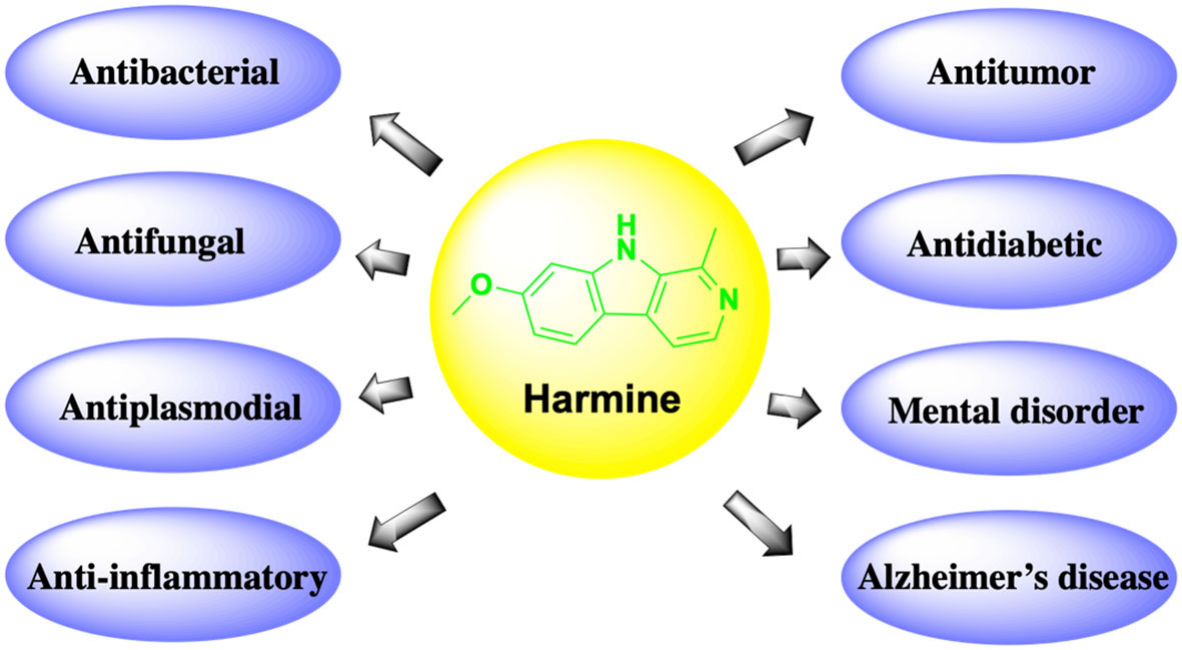
Pharmacological functions of harmine.

Harmine exhibits various pharmacological activities, including anti-inflammatory, antidiabetic, antifungal, antiplasmodial, and antitumor effects (**Figure 1**). Recently, it was reported that harmine and its derivatives could be drug candidates for the treatment of Alzheimer’s disease (7); additionally, they have been found to exhibit beneficial effects in patients with mental health disorders (8). Harmine can be used alone as a new anticancer drug or as an adjuvant treatment for cancer. However, due to its neurotoxic effects, various new derivatives have been developed to enhance efficacy and reduce toxicity. In this paper, we review the latest research on the effects of harmine and its derivatives on tumors *in vitro* and *in vivo*. In addition, we explore the regulatory mechanisms of various signaling pathways and molecular targets involved in the oncogenic effects of harmine, which have strong clinical potential and plasticity in different cancer types.

## 2 Mechanism of action of harmine in cancer

Several studies have confirmed that harmine inhibits tumor development in a dose- and time-dependent manner in breast, pancreatic, glioblastoma, and gastric cancers (9). The mechanisms underlying these antitumor effects include the inhibition of epithelial-to-mesenchymal transition (EMT), inhibition of angiogenesis, promotion of apoptosis, and regulation of the cell cycle.

### 2.1 Harmine inhibits the EMT in tumor cells

**Figure 2:**
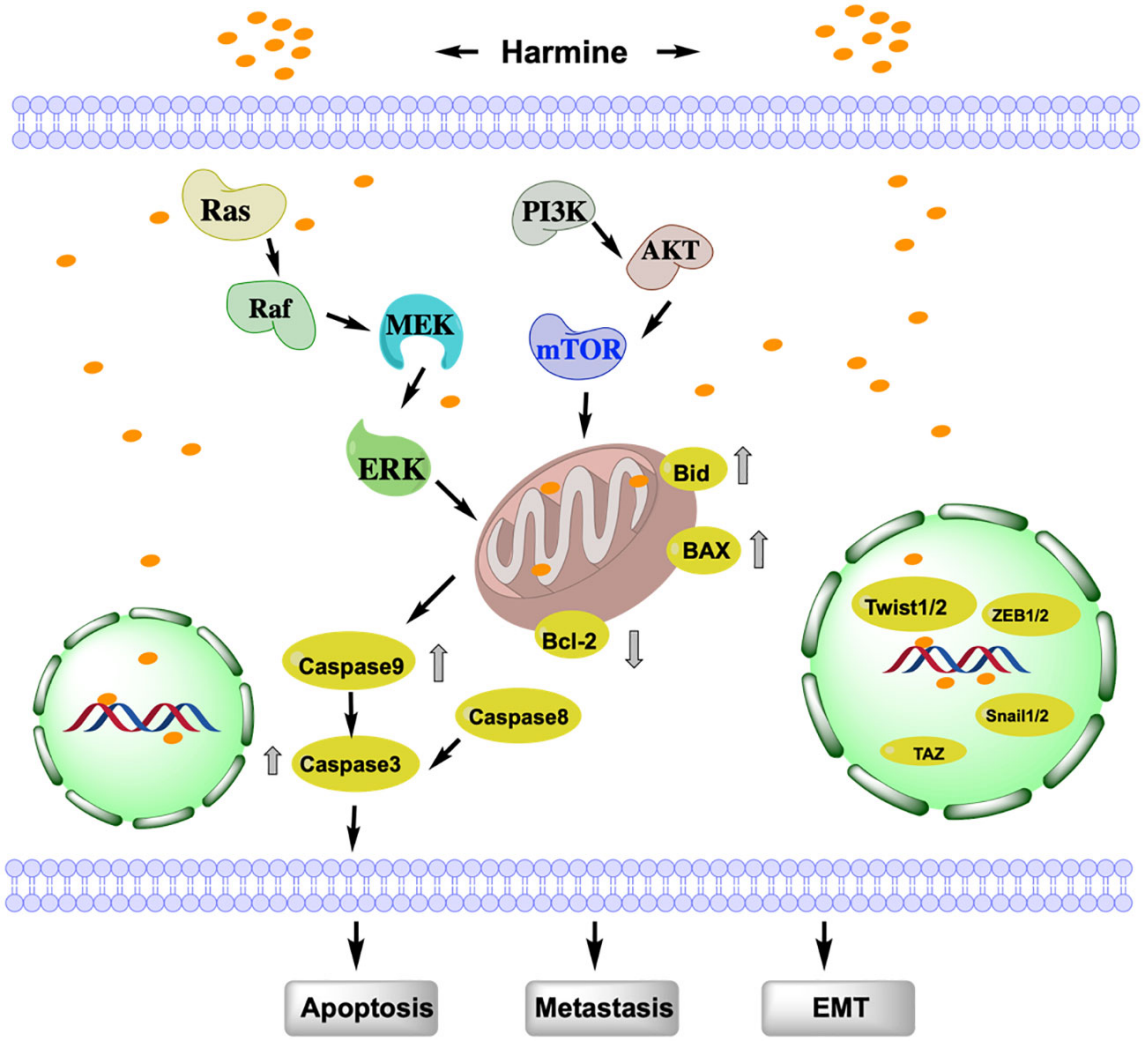
Study of the mechanism of action of harmine associated with EMT, metastasis, and apoptosis. Harmine induces tumor cell apoptosis via mitochondrial dependent- or independent-pathways and promotes EMT by binding to nuclear DNA. AKT, protein kinase B; Bax, Bcl-2 associated x protein; Bcl-2, B-cell lymphoma-2; Bid, BH3-interacting domain death agonist; EMT, Epithelial-Mesenchymal Transition; Erk, Extracellular signal-regulated kinase; MEK, mitogen-activated protein kinase kinase; mTOR, mammalian target of rapamycin; PI3K, phosphatidylinositol 3-kinase; Snail1/2, snail family transcriptional repressor 1/2; TAZ, transcriptional coactivator with PDZ-binding motif; Twist1/2, Twist-related protein 1/2; ZEB1/2, zinc finger E-box binding homeobox 1/2.

EMT is a process whereby epithelial cells separate from adjacent cells and acquire mesenchymal cell characteristics. The complex biological process of EMT is considered a critical indicator of the migratory ability of tumors. EMT in tumors is regulated by multiple signaling pathways; accordingly, targeting the EMT pathway is an important strategy for cancer treatment (10). Various studies have shown that harmine can inhibit EMT in tumor cells through different signaling pathways (**Figure 2**). Yao et al. discovered that harmine inhibits the invasion, metastasis, and EMT of breast cancer cells through the phosphatidylinositol 3-kinase (PI3K)/protein kinase B (AKT) signaling pathway (11). As the dose of harmine increases, the epithelial cell marker E-cadherin is upregulated in MCF-7 and MDA-MB-231 breast cancer cells. Meanwhile, the mesenchymal cell markers N-cadherin, vimentin, and fibronectin are downregulated. Additionally, the morphological changes in EMT-like cells are significantly suppressed.

Studies have shown that transcriptional co-activator with PDZ-binding motif (TAZ), which possesses a PDZ-binding domain, is overexpressed in tumors and is closely associated with tumor EMT (12). He et al. found that the overexpression of *TAZ* in breast cancer cells promotes the EMT, proliferation, invasion, and metastasis of tumor cells, whereas *TAZ* knockout inhibits the EMT, migration, and invasion of breast cancer cells. Harmine inhibits the EMT, invasion, and metastasis of breast cancer cells by targeting *TAZ* (13). In addition, harmine reduces the expression of *TAZ* in breast cancer cells, thereby inhibiting the proliferation, metastasis, and growth of tumor cells in subcutaneous xenografts in nude mice (14). Furthermore, harmine increases the phosphorylation levels of *TAZ* and inhibits its nuclear localization, thereby suppressing the EMT, metastasis, and invasion of MDA-MB-231 and MCF-7 breast cancer cells.

The transcription factor *Twist1*, belonging to the Twist family, which encodes a basic helix-loop-helix DNA-binding domain, is a key transcription factor that promotes EMT and cancer metastasis (15). *Twist1* is highly expressed in various cancers; it promotes tumor cell EMT and invasion by activating EMT-related target genes, such as *Bmi1, ZEB2*, and *Snail*, which enhance cell differentiation and migration rates (16). Yochum et al. discovered that harmine induces the proteasome-mediated degradation of *Twist1* in human and mouse breast cancer cells, thereby inhibiting the EMT in breast cancer cells (17). Dimerization of *Twist1* is critical for its function and stability. Compared to that of the Twist1-Twist1 homodimer, harmine preferentially promotes the degradation of the Twist1-E2A heterodimer in non-small cell lung cancer (NSCLC) cells and targets the Twist1-E2A heterodimer to exert cytotoxicity.

### 2.2 Harmine inhibits tumor invasion and migration by regulating angiogenesis

Metastasis is an important characteristic of malignant tumors, and angiogenesis is a crucial component of tumor metastasis. Accordingly, targeting angiogenesis is an effective strategy for inhibiting tumor metastasis. Cai et al. found that harmine inhibits bladder tumor invasion and migration by suppressing angiogenesis (18). The migration, proliferation, and lumen formation in endothelial cells during angiogenesis are mainly mediated by vascular endothelial growth factor (VEGF) and its downstream receptor, vascular endothelial growth factor receptor 2 (VEGFR-2) (19). Harmine inhibits angiogenesis by suppressing the phosphorylation of VEGFR-2 at *Tyr1175* in endothelial cells. High expression of matrix metalloproteinases (MMPs) is closely related to tumor angiogenesis, tumor cell escapes from blood vessels, and distant metastasis. MMPs play a crucial role in multiple extracellular and intracellular processes (20). Zhu et al. found that harmine inhibits the migration, invasion, and activity of glioma cells in a xenograft nude mouse subcutaneous tumor model by suppressing the expression of MMP2, MMP9, and VEGF (21). Gao et al. found that harmine inhibits the expression of VEGF, MMP2, and MMP9 in ovarian cancer SKOV-3 cells, thereby suppressing cell migration (22). Harmine also suppresses the migration and invasion of gastric cells by inhibiting MMP2 activity (23). Chin et al. discovered that harmine can serve as a specific inhibitor of MMP3 by targeting its enzymatic active site. The N2 bond of harmine binds specifically to the Zn^2+^ ion at the active site of MMP3, the methyl group at C1 interacts with His 205 and His 211, and the phenyl ring interacts with Val 163 at the active site, thereby inhibiting MMP3 activity. This effectively ameliorates the MMP3-mediated progression of malignant tumors (20).

### 2.3 Harmine induces tumor cell apoptosis through the PI3K/Akt/mammalian target of rapamycin axis

The apoptosis pathway of harmine can be divided into the death receptor (extrinsic) and mitochondrial (intrinsic) pathways (**Figure 2**). The extrinsic pathway involves the sequential activation of Caspase-8 and Caspase-3. The intrinsic pathway is activated by endogenous stimuli, leading to the release of cytochrome c and the activation of Caspase-9, which in turn, activates Caspase-3 (24); the anti-apoptotic protein B-cell lymphoma-2 (Bcl-2), pro-apoptotic proteins Bcl-2 associated x protein (Bax), and BH3-interacting domain death agonist (Bid) regulate this pathway (25). B16F10 cells treated with harmine exhibit characteristics of apoptosis, such as nuclear fragmentation, appearance of apoptotic bodies, and DNA ladder formation. The expression of Bcl-2 is reduced, while the levels of Bax, Caspase-3, 9, 8, and Bid are increased, indicating that harmine can induce apoptosis in melanoma B16F10 cells by simultaneously affecting both the intrinsic and extrinsic pathways (26). Furthermore, harmine induces apoptosis in thyroid cancer (27), liver cancer (28), and neuroblastoma cells (29) by regulating the Bcl-2/Bax ratio via the mitochondrial pathway. With increasing doses of harmine, Bax is upregulated in breast cancer cells, whereas Bcl-2, p-AKT, p-mTOR, and p-Erk are downregulated. However, no significant changes are observed in the expression of total AKT, mTOR, and Erk. This indicates that harmine induces apoptosis in breast cancer cells by inhibiting the PI3K/AKT/mTOR signaling pathway (29). Uhl et al. found that harmine induces cell shrinkage, reduces the mitochondrial membrane potential, and upregulates Caspase 3 and the autophagy-related proteins Beclin-1 and LC3 in gastric cancer cells. This mechanism of promoting cell apoptosis and autophagy is also achieved through the inhibition of the Akt/mTOR pathway (30). Harmine is also known to participate in tumor cell apoptosis and autophagy by regulating other mechanisms. In melanoma, harmine regulates pro-inflammatory cytokines, including tumor necrosis factor-α, interleukin (IL)-1β, IL-6, and granulocyte macrophage colony-stimulating factor, to promote tumor cell apoptosis (26). Harmine also induces endoplasmic reticulum stress and inhibits autophagy, which is regulated by the mTOR pathway, to suppress the malignant phenotype of esophageal squamous cell carcinoma cells (31).

### 2.4 Harmine inhibits tumor proliferation by regulating the cell cycle

**Figure 3:**
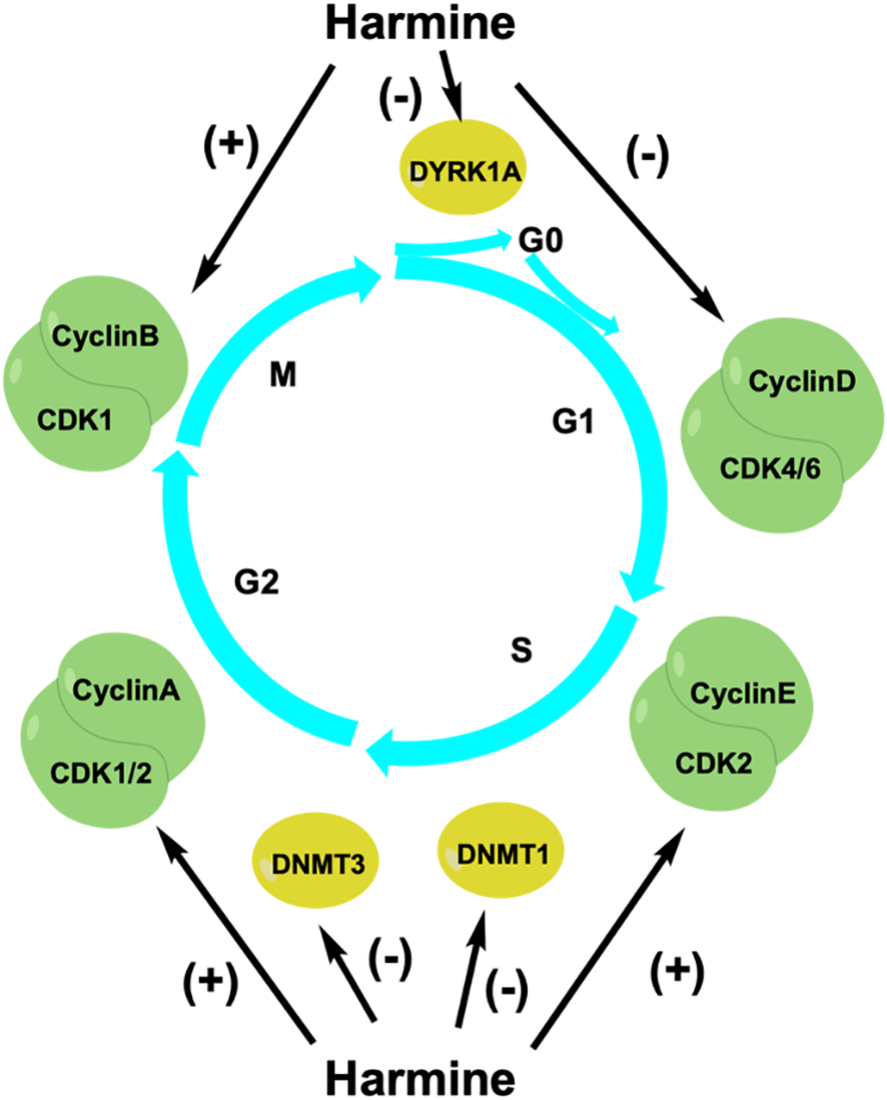
Harmine regulates the cell cycle to inhibit tumor cell growth. CDKs, cyclin-dependent kinases; DNMTs, DNA methyltransferases; DYRK1A, dual-specificity tyrosine phosphorylation-regulated kinase 1A.

The cell cycle is a highly regulated process that promotes cell growth, replication of genetic material, and cell division. Cyclins, cyclin-dependent kinases (CDKs), and CDK inhibitors (CKIs) are important regulators of this process (32). Studies have shown that harmine can inhibit tumor proliferation by regulating the cell cycle (**Figure 3**). Liu et al. found that harmine induced cell cycle arrest and inhibited the proliferation of colon cancer cells (33). Harmine treatment reduced cyclin D1 expression in colon cancer SW620 cells. Additionally, the expression of cyclins A, E2, and B1 was increased, leading to a decrease in the proportion of cells in the G0/G1 phase and an increase in the proportion of cells in the S and G2/M phases, thereby controlling cell proliferation. Cao et al. discovered that harmine induced cell arrest in the S and G2/M phases, thereby inhibiting the proliferation of liver cancer HepG2 cells (28). Shen et al. found that harmine selectively induced cell cycle arrest at the G1/S phase and inhibited the proliferation of NSCLC cells (34). Wu et al. found that harmine promoted the expression of cyclin B1, leading to cell cycle arrest at the G2/M phase and inhibition of the proliferation of pancreatic cancer PANC-1 and bxPC-3 cells (35). Studies have shown that dual-specificity tyrosine phosphorylation-regulated kinase 1A (DYRK1A) is a potential cancer therapeutic target, as it regulates the cell cycle by influencing the expression of oncogenes and tumor suppressor genes (36). DYRK1A specifically phosphorylates *LIN52* at serine 28 to regulate transition from the G0-G1 phase of the cell cycle, leading the cell to enter into a quiescent state (37). Francesco et al. discovered that harmine could serve as a specific inhibitor of DYRK1A to block the cell cycle and effectively reduce the number of cancer cells in both the quiescent and invasive states (38).

The balanced maintenance of DNA methylation during the cell cycle is important for the replication of genetic material and cell division. DNA methylation is a process wherein a methyl group from S-adenosylmethionine (SAM) is transferred by DNA methyltransferases (DNMTs) to the fifth cytosine carbon atom in CpG dinucleotide-rich regions. The main DNMTs in eukaryotic cells are DNMT1, DNMT3A, and DNMT3B (39). Aberrant hypermethylation of CpG-rich regions upstream of tumor suppressor genes leads to the recruitment of inhibitory complexes and transcriptional inactivation, resulting in abnormal cellular functions. Arezoo et al. found that harmine reduced the expression of DNMT1 in leukemia NB4 cells, leading to hypomethylation of the p15INK4b promoter of the CKI family and inducing cell cycle arrest at the G1 phase to inhibit cell proliferation (40). Harmine does not affect the mRNA expression of DNMT3A and DNMT3B but rather directly inhibits their activity to suppress prostate cancer cell proliferation. Cho et al. found that harmine inhibited prostate cancer cell growth by blocking mammalian DNMT activity. Harmine competitively binds to the SAM-binding site of the adenine domain in DNMT3B, thereby inhibiting the activity of the DNMT3B-3L complex and limiting prostate cancer cell proliferation (41). Therefore, harmine can serve as an important cell cycle regulator that inhibits tumor cell proliferation and exhibits antitumor activity. In addition to the aforementioned mechanisms, many new antitumor mechanisms have been discovered.

## 3 Synergistic anticancer effect of harmine combined with chemotherapeutic drugs

Treatment resistance is a major cause of cancer recurrence in the majority of patients with cancer, posing a significant challenge in managing those seeking to improve clinical outcomes. Research has shown that harmine exhibits potent synergistic effects when used in combination with various chemotherapeutic drugs. Harmine enhances the cytotoxicity of gemcitabine against pancreatic cancer cells and induces apoptosis. It can also act as a sensitizer of docetaxel, thereby enhancing its inhibitory effect against breast cancer cells (42). Combining harmine with a specific inhibitor of PI3K/Akt, such as LY294002, significantly increases its cytotoxicity in gastric cancer cells (30). Tribbles homologue 2 (TRIB2) is a protein that promotes the development of resistance to anticancer drugs and is an important driving factor for the development of resistance to multiple anticancer drugs. TRIB2 promotes AKT activity, leading to inactivation of the forkhead box, subgroup O (FOXO) transcription factor, which mediates the cellular response to anticancer drugs. Harmine can reverse the gene expression profile of TRIB2, promote the nuclear translocation of FOXO, and induce the transcription of FOXO target genes. This may weaken the TRIB2-mediated therapeutic resistance in human cancer cells (43). In addition, harmine can counteract regorafenib resistance in tumors by inhibiting the activation of the AKT pathway through the inhibition of DYRK1A activity (44). DYRK1A-mediated aberrant activation of the AKT pathway plays a crucial role in drug sensitivity during cancer treatment. During acquired resistance to regorafenib, harmine, in combination with regorafenib, significantly inhibits the proliferation of liver cancer HepG2 and Hep3B cells, promotes cell apoptosis, and upregulates p-AKT compared to single-drug treatment. High expression of the transcription factor *STAT3* in various cancer types is closely associated with resistance to chemotherapeutic drugs. Patients with wild-type *EGFR* exhibit resistance to AZD9291 treatment. Harmine sensitizes wild-type *EGFR* NSCLC cells to AZD9291 treatment by inhibiting DYRK1A activity and suppressing *STAT3* expression (45).

Harmine also serves as a radiosensitizer. Lan et al. found that a combination of harmine administration and radiotherapy increased the frequency of DNA double-stranded breaks and impaired homologous recombination in pancreatic cancer cells, thereby promoting cancer cell death. This provides a new strategy for overcoming the tolerance of pancreatic cancer cells to radiotherapy (46). Moreover, the combined use of harmine and paclitaxel can effectively induce apoptosis in gastric cancer cells (47, 48). Interestingly, harmine can also enhance therapeutic efficacy against melanoma in mice by modulating the immune microenvironment and synergizing with immunotherapy drugs. Harmine can upregulate the presentation of major histocompatibility complex class I (MHC-I) I-dependent antigens and the immune cell marker CD8 in melanoma cells. When combined with anti-PD1/PD-L1 therapy, it can improve the survival of cancer patients with defective MHC-I expression (49). The anticancer effects of the combination of harmine with radiotherapy and chemotherapy show promise for application in eliminating the resistance of patients with cancer during clinical treatment.

## 4 Structural modification, efficiency enhancement, and toxicity reduction of harmine

Camel Peony seeds have a wide range of antitumor effects, but they exhibit certain toxic side effects, associated with high doses and long-term exposure, which may cause serious renal and liver toxicity. The symptoms are mainly neurological (34.4%), gastrointestinal (31.9%), and cardiovascular (15.8%), with neurological toxicity being the most prominent (4, 50). β-Carboline compounds are the most active and abundant components in Camel Peony, and evidence suggests that these compounds are highly neurotoxic. β-Carboline compounds can stimulate the central nervous system by inhibiting the metabolism of amine neurotransmitters or directly interacting with specific receptors and are associated with the onset of Parkinson’s disease (51). The antitumor activity of β-carboline alkaloids, especially harmine, can be improved through structural modification or nanotechnology while simultaneously reducing their toxic side effects, which has important translational and clinical significance (52). Research has shown that the activity of harmine is related to the large hydrophobic groups present at positions 2, 7, and 9 in its ring. Modifications at positions 2 and 9 can enhance antitumor activity, and the insertion of long-chain substituents at position 7 can reduce neurotoxicity (53). Accordingly, the structural modification of harmine mainly focuses on these three sites (**Table 1**).

**Table 1:** Novel derivatives of harmine and their bioactivity in tumor cells 
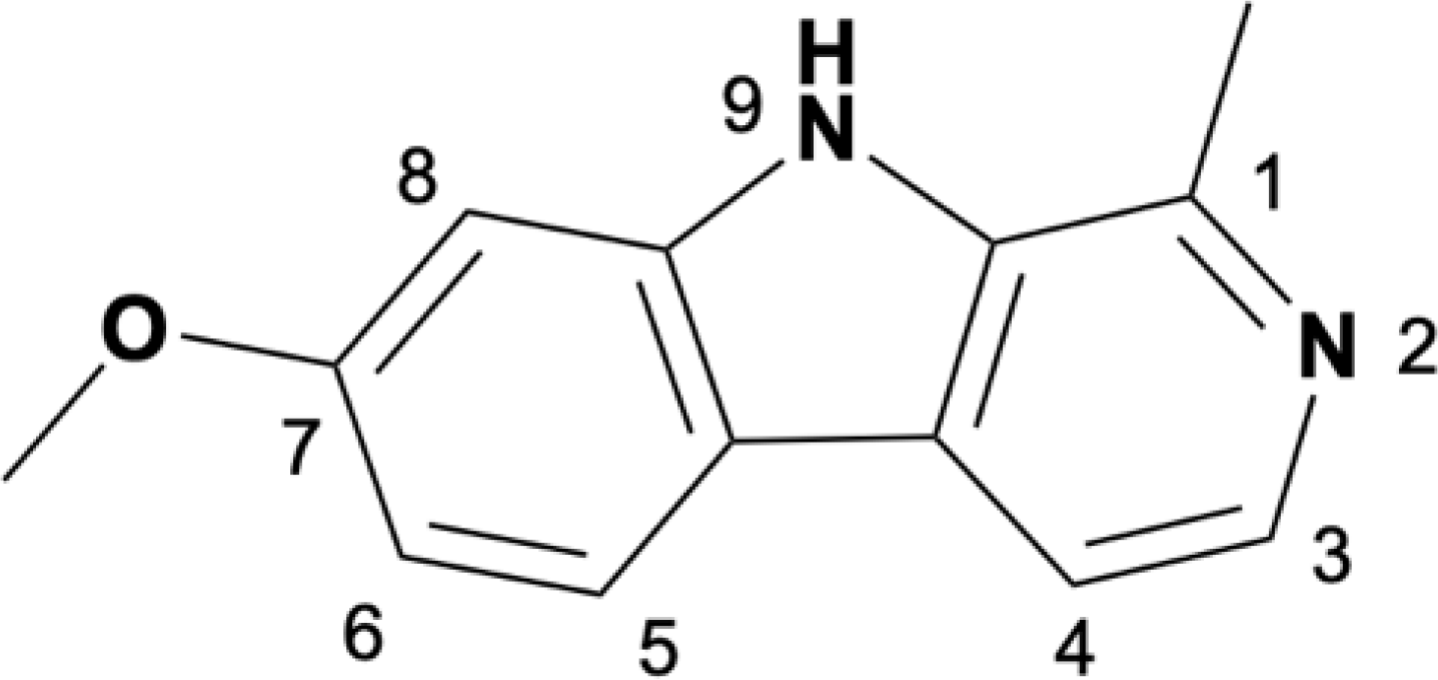
.

Compound	Position	Chemical group	Antitumor activity	Acute toxicity	Neurotoxicity	Ref
8	9	N9-benzyl	↑	↓	↓	54
5a	2,7,9	N2-benzyl, 7-benzyl, N9-benzyl	↑	ND^a^	ND	55
5p	7,9	7-alkyl, N9-alkyl	↑	↓	↓	56
3c	1,2,9	1-benzylindine, N2-benzyl, N9-alkyl	↑	↓	↓	57
10	2,7,9	N2-benzyl, 7-furoxan, N9-alkyl	↑	↓	↓	58
27	9	N9-HDACIs	↑	ND	ND	60
G11	1	1-chalcone	↑	↓	↓	61
CM16	2,7,9	N2-alkyl, 7-alkyl, N9-alkyl	↑	ND	ND	62
10f	2,9	2-benzyl, N9-alkyl	↑	ND	ND	63
11d	9	N9-acylation	↑	ND	ND	64
36	7	7-Ferrocene	↑	ND	ND	65

a ND, not tested. ↑, means increase; ↓, means decrease.

Analysis of the structure-activity relationship of harmine revealed that the formate ester substituent at position 3 of the tricyclic skeleton can reduce neurotoxicity, and the short-chain alkyl or aryl substituent at position 9 can enhance its antitumor activity. In 2005, Chen et al. found that compound 8 exhibited the highest cytotoxicity in HepG2 cells, while modification at position 9 increased its antitumor activity (54). To overcome the intrinsic resistance of cancer cells to apoptosis, Fred́eŕick et al. synthesized a series of novel harmine derivatives, and compound 5a demonstrated approximately 100 times more activity than harmine. They also found that highly active *β*-carbolines were more lipophilic and larger than the less active compounds (55). Cao et al. found that the methoxy group at position 7 might be the key factor causing neurotoxicity. Replacing the 7-methoxy group with a bulky alkoxy moiety significantly reduced or even eliminated the neurotoxicity caused by harmine. Compound 5p exhibited the highest antitumor activity with low acute toxicity in mice (56). The authors later found that introducing an appropriate substituent at position 9 significantly enhanced the antitumor activity of this compound both *in vitro* and *in vivo*. Compound 3c exhibited significant cytotoxic effects against tumor cells and low toxicity against normal cells. A mechanistic study revealed that compound 3c induced tumor cell apoptosis via the PI3K/AKT signaling pathway (57).

Li et al. synthesized harmine derivatives containing nitric oxide that release lead compounds. After conducting *in vitro* cytotoxicity evaluations using five human cancer cell lines, compound 10 was found to have significantly improved antitumor cell proliferation activity, compared to harmine, and exhibited favorable plasma stability. The results of acute toxicity studies in mice suggested that compound 10 significantly reduced acute toxicity in mice when compared to harmine (58). Shankaraiah et al. synthesized a series of novel C3-tethered1,2,3-triazolo-β-Carboline derivatives that exhibited favorable antitumor activities and DNA-binding affinity *in vitro* (59). Lu et al. designed harmine-based dual inhibitors that target histone deacetylases (HDAC) and DNA. They introduced a zinc-binding group at position 9 and explored its action mechanism in inhibiting HDAC activity using derivatives of dehydroalkaline substituted at positions 3 and 7. They found that compound 27 could bind to DNA and exhibited the strongest antitumor activity against cancer cells while displaying low toxicity toward normal cells (60).

Guo et al. synthesized a series of β-carboline derivatives by introducing a chalcone moiety into harmine. These novel compounds could act as Topo I inhibitors that affect DNA synthesis and exhibited favorable antitumor activities both *in vitro* and *in vivo* (61). Carvalho et al. found that 2,7,9-trisubstituted CM16 displayed anticancer effects *in vitro* by penetrating the perinuclear region of the endoplasmic reticulum and targeting translation initiation sites, thereby reducing protein synthesis in a time- and concentration-dependent manner. *In vitro* cell viability experiments have also shown that CM16 is more effective than harmine at inhibiting tumor cell proliferation (62). Geng et al. synthesized 2,9-disubstituted harmine derivatives, of which 10f exhibited significant growth inhibition in four cancer cell lines. They also found that 10f induced apoptosis and inhibited autophagy in a dose- and time-dependent manner (63). A series of N9-substituted harmine derivatives were synthesized by introducing haloalkyl or benzenesulfonyl groups. All the synthesized compounds exhibited higher anticancer activities than the parent compound. Compound 11d exhibited the highest anticancer activity and induced apoptosis via cell cycle arrest (64). Poje et al. designed and synthesized 18 novel compounds by combining harmine and ferrocene. Compound 36 exhibited a better antitumor activity than its parent compound *in vitro* (65).

## 5 Conclusion and perspectives

As a main active ingredient in traditional Chinese herbal medicines, harmine possesses excellent antitumor activity and can inhibit tumor proliferation, invasion, and migration through various pathways. Harmine also reduces drug resistance when used in combination with chemotherapeutic drugs. More derivatives with higher anticancer activities and lower toxicities have been developed by modifying the structure of harmine. Although most compounds exhibit favorable activity and stability, some derivatives have low oral bioavailability. Novel harmine derivatives may have significant clinical application prospects, but further validation based on drug activity, acute toxicity, and other aspects is necessary. In summary, further research on the mechanisms of action of harmine in various tumors is needed for improving drug activity research and clinical translation.

## Author contributions

YH: Conceptualization, Writing – original draft, Writing – review & editing. XY: Writing – original draft. LY: Writing – original draft. GX: Conceptualization, Writing – review & editing. QW: Writing – review & editing. ZH: Visualization, Writing – original draft, Writing – review & editing. HC: Writing – review & editing.
